# Older Adult Fall Risk Prediction with Deep Learning and Timed Up and Go (TUG) Test Data

**DOI:** 10.3390/bioengineering11101000

**Published:** 2024-10-05

**Authors:** Josu Maiora, Chloe Rezola-Pardo, Guillermo García, Begoña Sanz, Manuel Graña

**Affiliations:** 1 Electronic Technology Department, Faculty of Engineering of Gipuzkoa, University of the Basque Country, 20018 San Sebastian, Spain; 2 Computational Intelligence Group, Department of CCIA, University of the Basque Country, 20018 San Sebastian, Spain; manuel.grana@ehu.eus; 3 Department of Physiology, University of the Basque Country, 48940 Leioa, Spain; chloe.rezola@ehu.eus (C.R.-P.); mariabegona.sanz@ehu.eus (B.S.); 4 Systems and Automation Department, Faculty of Engineering of Gipuzkoa, University of the Basque Country, 20018 San Sebastian, Spain; g.garcia@ehu.eus; 5 Biobizkaia Health Research Institute, 48903 Barakaldo, Spain

**Keywords:** inertial sensors, fall prediction, fall risk assessment, deep learning, machine learning

## Abstract

Falls are a major health hazard for older adults; therefore, in the context of an aging population, predicting the risk of a patient suffering falls in the near future is of great impact for health care systems. Currently, the standard prospective fall risk assessment instrument relies on a set of clinical and functional mobility assessment tools, one of them being the Timed Up and Go (TUG) test. Recently, wearable inertial measurement units (IMUs) have been proposed to capture motion data that would allow for the building of estimates of fall risk. The hypothesis of this study is that the data gathered from IMU readings while the patient is performing the TUG test can be used to build a predictive model that would provide an estimate of the probability of suffering a fall in the near future, i.e., assessing prospective fall risk. This study applies deep learning convolutional neural networks (CNN) and recurrent neural networks (RNN) to build such predictive models based on features extracted from IMU data acquired during TUG test realizations. Data were obtained from a cohort of 106 older adults wearing wireless IMU sensors with sampling frequencies of 100 Hz while performing the TUG test. The dependent variable is a binary variable that is true if the patient suffered a fall in the six-month follow-up period. This variable was used as the output variable for the supervised training and validations of the deep learning architectures and competing machine learning approaches. A hold-out validation process using 75 subjects for training and 31 subjects for testing was repeated one hundred times to obtain robust estimations of model performances At each repetition, 5-fold cross-validation was carried out to select the best model over the training subset. Best results were achieved by a bidirectional long short-term memory (BLSTM), obtaining an accuracy of 0.83 and AUC of 0.73 with good sensitivity and specificity values.

## 1. Introduction

Older people suffering falls often require medical attention [1,2], hence falls are becoming a major public health problem due to the increasing aging of the population. The rising incidence of accidental falls has a great economic impact on health care systems and society: 20–30% of falls lead to mild to severe injuries, and falls were the underlying cause of 10–15% of all emergency department visits of older people in the United Kingdom in 1999 [3]; these figures are growing, with the population aging since then. Moreover, falls often cause mobility impairments that lead to dependency for activities of daily living, along with psychological consequences such as anxiety and fear for future falls [4,5]. According to the World Health Organization, the approximate worldwide yearly incidence of falls for people over 65 years old is 28–35%, increasing to 32–42% for people aged over 70 years [6]. In particular, older adults living in nursing homes are especially prone to falling. In fact, the fall incidence in this population is three times that of older people living in the community [7]. The financial toll from older adult falls in the United States the medical costs attributed to nonfatal and fatal falls amounts to approximately $50 billion every year [8]. Therefore, fall prevention in older adults is of utmost socioeconomic importance.

To this end, clinical questionnaires and clinical assessment-based fall risk prediction tools have been proposed reporting a wide range in performance scores (sensitivity in the range 14–94%, specificity in the range 38–100%) [9]. Additionally, fall risk assessment protocols like the STEADI (stopping elderly accidents, deaths, and injuries) proposed by the Centers for Disease Control (CDC) rely on functional mobility assessment tools in the form of questionnaires, physical tests, gait analysis, and physical activity measurements [10]. Some of the most widely used assessment tools are the Timed Up and Go (TUG) test [11], the Tinetti Assessment Tool [12], the STRATIFY score [13], and the Five-Times-Sit-to-Stand (FTSS) test [14]. Specifically, the TUG test has proven valuable in early assessment of balance and mobility [15,16,17]. However, all of these tools are in fact used qualitatively by the clinician trying to assess prospective fall risk.

The main hypothesis of this study is that the information extracted from IMU readings during the realization of the TUG test can be used to build predictive models that provide an estimate of the probability of the patient suffering a fall in the near future. In other words, this information may be used for quantitative and predictive fall risk assessment. This information would be of great importance to guide fall prevention for older adults, and especially for those living in nursing homes due to their greater fall incidence.

The paper reports two computational experiments. The first corresponds to application of supervised machine learning algorithms to some descriptive variables of the TUG test phases. The second corresponds to the application of deep learning architectures over the raw data of the IMU wearable.

The contributions of this paper are the following: (a) the collection of a dataset of IMU readings while a large number of subjects are realizing a TUG test whose {F,NF} labels are generated in a follow-up period of 6 months; (b) the proposal of deep learning architectures to deal with this prediction problem; (c) the proposal of feature extraction processes and conventional machine learning for comparison with the deep learning approaches.

## 2. Materials and Methods

Recent surveys on the application of machine learning methods for prospective and retrospective discrimination between patients who experience falls, i.e., fallers, (F) from non-fallers (NF) using IMU information report widely different predictive performance results (accuracy: 62–100%, sensitivity: 55–99%, specificity: 35–100%) in populations over 65 years old [18,19,20,21]. These surveys also report a large heterogeneity of sensor placement, tasks assessed, and sensor features. Specifically, some authors found that data from wearable IMU sensors add meaningful information to the TUG test [22].

Deep learning architectures have been applied successfully in many areas of computer vision [23], medical image analysis [24], assisted/autonomous driving [25], and machine anomaly monitoring [26], to name a few applications. Deep learning has already been applied to the classification of IMU sensor data [27,28,29,30] for human activity recognition. However, multiple data sources and adequate assessment tests are necessary to generalize fall risk predictions. Nait Aicha et al. [31] compared deep learning approaches to traditional machine learning methods to model fall risk on the basis of daily life body trunk accelerometer data. They acquired data of participants wearing a triaxial accelerometer for 1 week. They evaluated convolutional neural network (CNN), the long short-term memory (LSTM) model, and a combination of both which they refer to as the “ConvLSTM”, reporting good results in modeling the training data, but it generalized poorly over new subjects and the relatively long period during which subjects must wear the inertial sensor is a barrier to its implementation.

Due to the multidimensional nature of the risk of falls in older adults, there is no single ideal tool that performs a perfect risk assessment in any context. For this reason, the simultaneous application of multiple tools is recommended [32].

The present article presents a secondary analysis of two single-blinded and multicenter randomized controlled trials that were registered with codes [ACTRN12618000536268; NCT03996083] whose primary outcomes have previously been published [33,34]. This study includes 106 subjects (68 women and 38 men) from 9 long-term nursing homes (LTNHs) (Gipuzkoa, Basque Country, Spain). Subject’s ages ranged from 70 to 104 years old and their physical and cognitive characteristics were described previously [35]. After providing written consent, participants performed the TUG test twice wearing a wireless inertial sensor (G-Walk, BTS Bioengineering Corp., Milan, Italy) and the best (fastest) trial was selected. This sensor was placed on the lower back area in order to quantify the center of mass movement. This study was approved by the Committee on Ethics in Research at the University of the Basque Country (Humans Committee Code M10/2016/105). All feature extraction and classification cross-validation was carried out in Matlab 2022b using wavelet, statistics and machine learning, and deep learning toolboxes. For performance evaluation we split the data in 5 groups and in each iteration, we hold out one group/fold and train the algorithms in the remaining 4 groups. We perform this method to obtain a less biased model than other methods, such as a simple train/test split. This process is carried out for all evaluated classifiers and feature extraction techniques. A fall was defined as an unintentional event in which the person comes to rest on the ground, not as a result of an epilectic seizure or an acute stroke [36]. Falls suffered by the residents are systematically detected and immediately recorded in the database by the staff of each nursing home. Information regarding residents who experienced any fall during 6-month follow-up period was extracted from the participant’s medical record as provided by the medical staff. Participants were labeled as faller (F) or non-faller (NF). The number of falls was not taken into consideration in the present study.

### 2.1. Data and Feature Extraction

#### 2.1.1. TUG Test Realization for Data Capture

**Figure 1:**
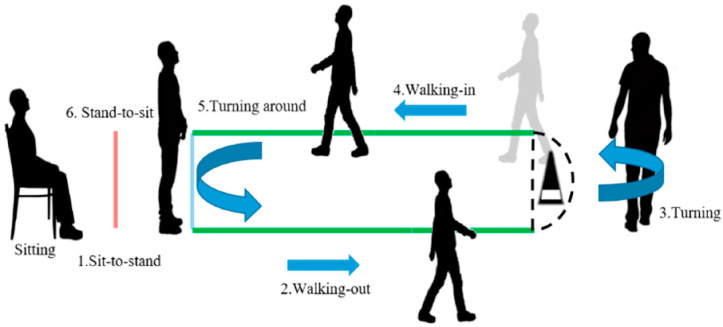
The process of the realization of the TUG test decomposed into six phases.

The TUG test process is decomposed into six phases, as shown in Figure 1, which are described as follows:

1. The time elapsed from the beginning of standing-up motion up to the instant when the subject stands up;
2. The time elapsed walking from the initial standing up position to the position where s/he starts turning down;
3. The time elapsed while turning down;
4. The time elapsed walking back to the chair from the end of the first turn to the beginning of the second turn;
5. The time elapsed turning to prepare to sit down, and
6. The time elapsed sitting down in the chair, completing the TUG test.

#### 2.1.2. Raw IMU Data and Labels

**Figure 2:**
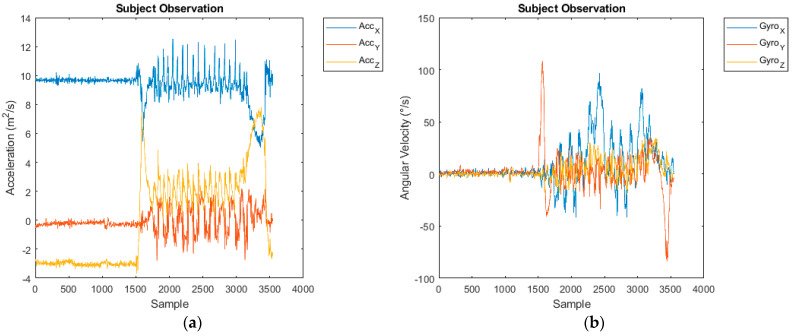
An instance of the readings of the G-walk during a TUG test realization shown as raw data plots: (**a**) triaxial accelerometer, and (**b**) triaxial gyroscope.

The G-Walk IMU sensor acquires acceleration, angular velocity and magnetic field data. Its components are a triaxial accelerometer (x, y, z), a triaxial gyroscope (x, y, z), and a triaxial magnetometer (roll, pitch, yaw). Sampling frequency was adjusted to 100 Hz. The accelerometer has a resolution of 16 bits per axe and its sensitivity was adjusted to 2 g. The gyroscope also has a resolution of 16 bits per axe and its sensitivity was adjusted to 2000°/s. The magnetometer has a resolution of 13 bits with a sensitivity of 1200 µT. Figure 2 shows example readings from the sensor.

**Figure 3:**
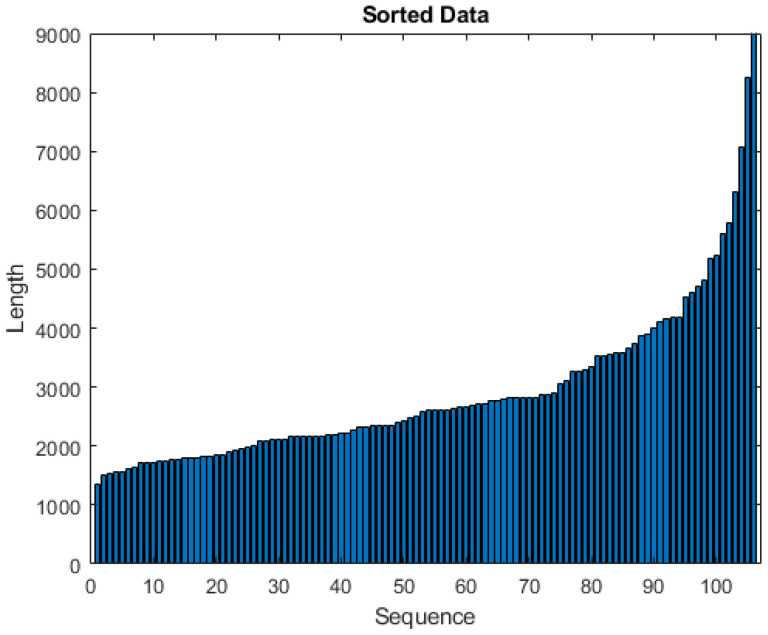
Number of samples per recorded IMU sequence during the realization of TUG tests sorted in ascending order.

We collected raw IMU data for each TUG test realization by a subject. Due to variability in the time taken to perform the TUG test, the number of samples per subject varies from 1364 to 9975 as shown in Figure 3. Additionally, data of patients suffering fall occurrences during a 6-month follow-up period were collected and provided to the researchers by the staff of the LTNHs. In this period, 21 subjects (19%) were labeled as fallers (F). This label data are used as the dependent variable in the training and validation of the classification algorithms, for both the deep learning networks and conventional machine learning approaches.

In the pre-processing steps, we remove the subjects with missing values of IMU sensors or without label information (faller/non-faller). Then, we sort the subjects regarding their number of samples, observing that the great majority of them have less than 5000 IMU data samples, and that those above this number could be considered outliers. However, these subjects are precisely the ones that have a higher fall risk. Consequently, we train our model with data from all the subjects.

The class imbalance in the dataset is moderate (ratio 1:5); however, conventional machine learning approaches are usually biased towards the majority class, which in this study is the non-fallers (NF) class, suffering from low sensitivity even when reporting high accuracy [37].

The norm of the 3D acceleration vectors is computed at each instant in order to obtain a scalar time series. In this way, significant changes in acceleration magnitude, which occur in events such as walking, turning, or getting up/sitting in the chair, are easily detected regardless of the orientation of the device.

#### 2.1.3. TUG Test Variables per Phase

We recorded spatiotemporal measurements of the IMU wearable sensor during TUG test realizations decomposed into standing phase, sitting phase, walking phases, and body trunk rotations (flexion and/or extension angle). These measurements are used as input variables by the conventional machine learning classification algorithms. Table 1 shows the maximum, minimum and average values of each of these parameters across subjects. The first group of variables are the duration of the different phases. During the “Sit to Stand” and “Stand to Sit” phases, we recorded the vertical, media-lateral, and anterior–posterior accelerations, as well as extension and bending angles. In the “Turning” phases, we recorded the angular accelerations. We recorded the duration of each activity phase for all subjects, computing the mean and variance of each of them.

**Figure 4:**
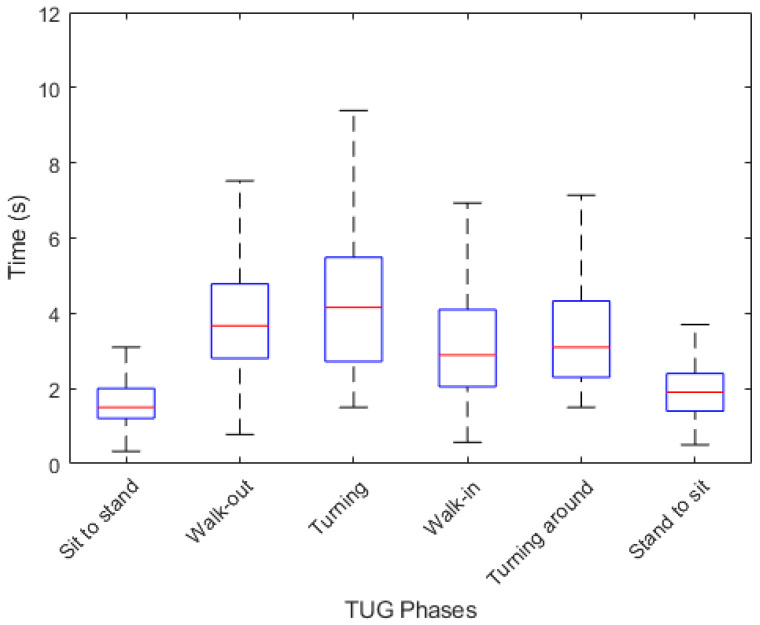
Box-plot of each phase duration in TUG test. The median, upper-lower quartiles and maximum-minimum values are shown.

**Figure 5:**
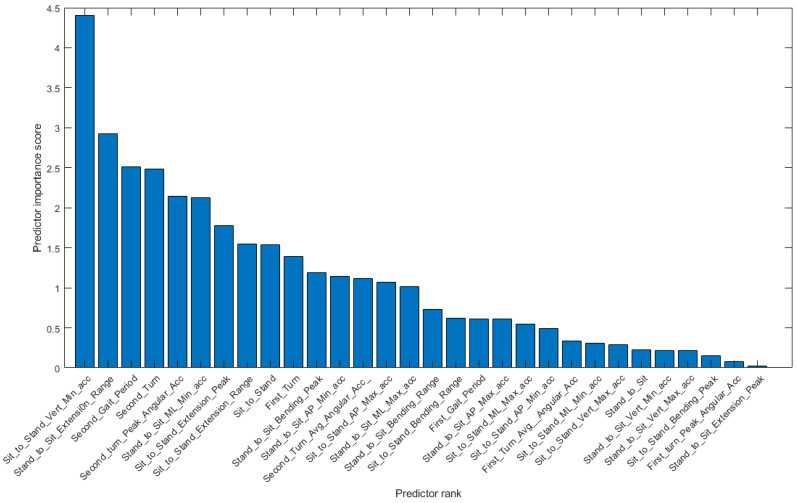
Univariate Chi-Square Test importance ranking of TUG test phase input variables used by conventional machine learning classifiers.

Figure 4 shows a box plot of the duration of each phase. The turning phase has the longest average duration followed by the walking out, turning around and walking-in phases. The sitting and standing activities have the shortest average durations. We compute the univariate Chi-Square Test [38] of each feature relative to the {F, NF} class label, obtaining the feature importance ranking shown in Figure 5.

#### 2.1.4. Wavelet Features

Wavelet Transforms (WT) are used to represent a signal in terms of localized basis functions called wavelets. WT use a wavelet function and a lowpass scaling function to generate low-variance representations of real-valued time series data at different time scales. The general formulation of the wavelet is like the following equation:

$$\gamma \left(s,\tau \right)=\frac{1}{\sqrt{s}}\int x\left(t\right)\cdot \psi \left(\frac{t-\tau }{s}\right)$$

Traditional frequency analysis methods such as the Fourier Transform yield only frequency-domain information without any indication of the temporal location/extent of a given frequency component. Wavelet transforms, on the other hand, provide both temporal and frequency information, as the basis functions it relies upon are localized in both time and frequency.

The IMU readings are transformed by the wavelet time scattering decomposition using the Gabor wavelet [39] that yields representations insensitive to translations in the input signal without sacrificing class discriminability and separate the data from different classes as far clear as possible. These wavelet features are obtained after applying the filter banks of the wavelet transform to our signals The scattering sequences are 38-by-1250, where 1250 is the number of time steps and 38 is the number of scattering scales. This matrix constitutes the input features for our 1-D CNN approach to fall risk prediction. Additionally, for we consider each element of the matrix as an independent feature. As a result, we receive 47,500 independent wavelet features with this decomposition. Due to the large number of features, we need to carry out a feature selection process to enhance the efficiency of the model. The importance of each wavelet feature to discriminate faller vs. non faller is evaluated by individual Chi-square tests [38]. Finally, we choose the 20 most significant wavelet features as the optimal ones. Increased number of wavelet features did not improve the classification performance.

### 2.2. Machine Learning

The fall risk assessment is stated as a binary classification problem, where the classes are {F, NF} labels assigned in the follow-up period after the IMU measurements (hence, we deal with a prospective problem).

#### 2.2.1. Conventional Machine Learning Algorithms

We have applied the following 5 conventional Machine Learning (ML) algorithms to classify the subjects according to their fall risk assessment: Random Forest (RF), Support Vector Machines (SVM), K nearest neighbors (KNN), Naive Bayes (NB). The hyper-parameters of the machine learning algorithms are set as follows: RF: #splits = 105, #learners = 30 SVM: quadratic kernel; KNN: K = 10; NB: Gaussian kernel. The implementations used are the standard ones provided in MATLAB. Conventional ML algorithms are applied over TUG test phase variables described in Table 1, because the raw IMU signals have an extremely large dimensionality to be used as inputs for the selected ML models.

#### 2.2.2. Deep Learning Neural Network Models

One of the most distinctive characteristics of deep learning approaches is that they learn a hierarchy of abstract representations from the raw data [40] overcoming the need to define and tune specific features for the problem at hand. In fact, most deep learning approaches are artificial neural networks, so that the term “deep” refers to the number of layers in the network—the more layers, the deeper the network. Two of the most popular deep learning networks are the convolutional neural network (CNN) [41] and the long short-term memory (LSTM) [42]. CNNs built up a hierarchy of convolution filters trained from the data. We use a specific brand of CNNs whose input data are extracted by means of Scattering Wavelet Transforms [42,43] in its 1D version.

An LSTM is good for classifying sequential and time-series data, when the prediction or output of the network must be based on a remembered sequence of data points. An LSTM is a type of recurrent neural network (RNN) [44] that can learn long-term dependencies between time steps of sequence data. Unlike a CNN, a LSTM can remember the state of the network between predictions [23]. The core components of a LSTM network are a sequence input layer and a LSTM layer. A sequence input layer incorporates time-series data into the network. A LSTM layer learns long-term dependencies between time steps of sequence data over time. The LSTM is trained on the raw IMU readings after computing the norms of the 3D vectors of each measure.

## 3. Results

We have performed four different computational experiments evaluating the different fall risk predictors’ performance in terms of accuracy, sensitivity, and specificity. In the case of raw data, we have also computed the area under the receiving operator curve (AUC). In all cases, we have carried out 100 repetitions of the holdout cross validation with 75 subjects for training and 31 for testing using stratified sampling in the sample extraction, and 5-fold cross-validation over the training set to select the best model for testing at each holdout repetition.

### 3.1. Conventional Machine Learning Classifiers

We have carried out two different computational experiments with conventional ML classifiers that will serve as benchmarks for the deep learning approaches. In the first experiment, we use as features the aggregated spatiotemporal measurements of the realizations of TUG test corresponding to standing phase, sitting phase, and rotations body trunk kinematics from Table 1. The results are shown in Table 2. We have carried out the classifier validation experiments over three distinct subsets of features: (a) the most important TUG phase descriptive variables selected by independent Chi-square tests, (b) the duration of each phase of the TUG test, and (c) the entire set of TUG phase descriptive variables. Results are rather poor for all models and features, with accuracy below 0.7, and sensitivity below 0.33.

In the second experiment, we apply the ML classifiers to the selection of the 20 most significant wavelet scattering features extracted from the magnitude of the acceleration signal. Results presented in Table 3 show significant improvement over results reported in Table 2. The increase in specificity may be due to the class-imbalance induced bias, while the naive Bayes approach achieves an average sensitivity of 0.52, which is the best result found.

### 3.2. Deep Learning Results

#### 3.2.1. CNN

We evaluate 1-D CNN using as inputs the wavelet scattering matrices computed over the acceleration magnitude. The scattering sequences are 38-by-1250 where 1250 is the number of time steps and 38 is the number of scattering paths. Results are shown in Table 4 for various selections of gradient descent optimization methods (RMSProp, SGDM, and Adam). Results improve over the ML conventional classifiers in terms of accuracy; however, they are not above of RF in terms of AUC, which for many authors is a more appropriate performance measure for class imbalanced datasets.

#### 3.2.2. LSTM

We evaluate LSTM deep learning algorithms over raw inertial sensor data (triaxial accelerometer, gyroscope and magnetometer). Both standard LSTM and bidirectional LSTM (BLSTM) were used as we have access to the entire sequence of data. We evaluated mini-batch sizes from 5 to 25 with number of hidden units set to 40 and a learning rate of 0.005. The best accuracy results were obtained for mini-batch sizes of 10, 11, and 15. To find the optimal number of hidden units, we set the mini-batch size to 11 and evaluated the accuracy beginning from 10 until 100 units using increments of 10. The best values are obtained for 40 hidden units. We chose a mini-batch size of 11. Subjects were ordered according to their number of samples and shuffle was disabled to reduce the “padding effect”.

**Figure 6:**
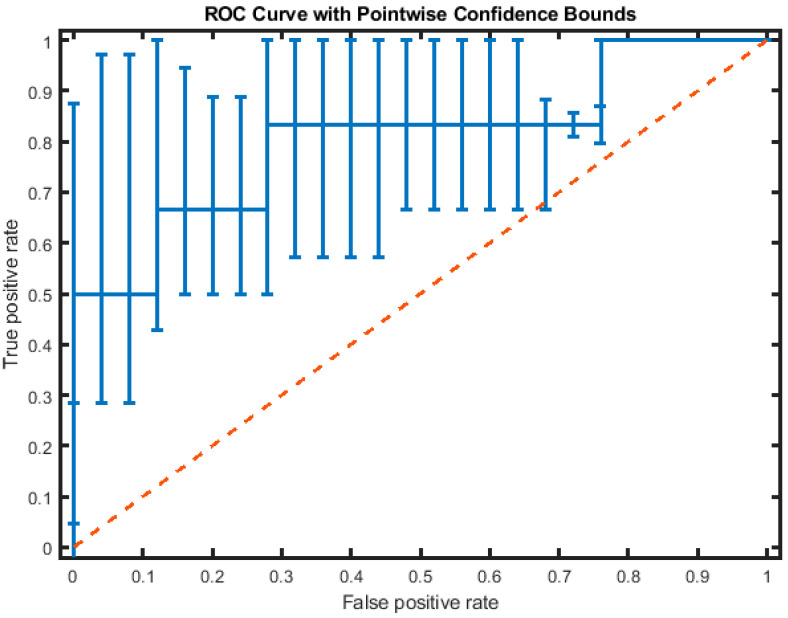
ROC curve with Point-wise Confidence Bounds of an instance of the 5-fold cross-validation of the BILSTM architecture. The dashed lines represent the chance ROC.

Table 5 shows the average test performance results after 100 repetitions of hold-out cross-validation of various LSTM architectures. We found that BLSTM performance measures are significantly better than standard LSTM results for every mini-batch size and the best size for BLSTM is ten. The BLSTM trained with SGDM significantly outperforms all other approaches in terms of sensitivity and AUC. Figure 6 shows the corresponding ROC curve with point-wise confidence bounds.

## 4. Discussion

In the present study, conventional machine learning classifiers and deep learning networks have been applied to prospective fall risk prediction over IMU sensor data captured during the realization of the TUG test for a cohort of older adults (N = 106, of which 21 are fallers). The hypothesis of this work is that processing these data with machine learning and deep learning approaches would allow prospective fall risk prediction. We have explored several signal features, including the raw signal, and several machine learning and deep learning approaches. The best results in terms of sensitivity (i.e., accurate prediction of fallers) have been obtained by the naive Bayes approach on wavelet scattering features (sensitivity = 0.52), and by the BLSTM trained with SGDM on the raw IMU signal data (0.50). We obtained high specificity in many instances; however, the cost of misclassification of a faller is higher than misclassification of a non-faller, hence sensitivity is a more relevant performance measure. It was argued that the ability of the TUG test to assess prospective fall risk was limited [14]; however, our results show that processing the IMU sensor data, that implicitly takes into account postural stability, gait, stride length, and sway, a fair prediction of fall risk can be achieved. In the future, we will be testing our approach in larger cohorts. Additionally, we will be exploring the application of Generative Adversarial Networks (GAN) for the enrichment of the faller class in order to obtain more balanced datasets for training and synthetic data generation techniques like SMOTE (Synthetic Minority Over-sampling Technique). We believe our results are promising and could contribute to fall prevention enhancement. This is important and would directly benefit older adults themselves, as those at risk of falling would be identified beforehand and it would enable the relevant entities to consider proper measures and to implement strategies to prevent falling, ultimately preserving their independence and reducing medical care costs.

## 5. Conclusions

Falls are among the most significant challenges faced by older adults, making their assessment and prevention critically important, particularly in the current demographic context. Although several tools exist for assessing fall risk, these are typically based on time, distance, or visual observation metrics. In fact, these tools are of qualitative nature helping to guide the medical staff assessment. Our approach, by contrast, leverages the large amount of information that can be collected by wearable IMU sensors on individuals being studied while performing the Timed Up and Go (TUG) test; specifically, we can use the raw data from the accelerometer, gyroscope, and magnetometer. Given the relatively high sampling frequency (100 samples per second), the duration of the test, and the three-dimensional data produced by each of the three sensors, a substantial volume of data are generated. The most effective way to analyze such data, with current technological capabilities, is through the application of artificial intelligence. The study includes 106 subjects (68 women and 38 men) from 9 long-term nursing homes (LTNHs). Upon comparing traditional machine learning methods with deep learning approaches, it was found that the latter yielded the most accurate results, specifically the BLSTM algorithm. We believe that our method complements traditional fall risk screening methods and adds valuable information to improve the assessment of subjects with frailty.

## Figures and Tables

**Table 1:** Descriptive statistics of the spatiotemporal measurements of the TUG test realizations corresponding to standing phase, sitting phase, and rotations body trunk kinematics (flexion and/or extension angle). Accelerations (acc) are measured in m/s^2^. Body trunk rotations are measured in degrees. Anterior–posterior (AP), Medio-Lateral (ML), and Vertical (Vert) axis accelerometer data are shown.

Variable	Max	Min	Average
Phase Duration			
Sit_to_Stand (s)	4.7	0.33	1.73
Walking_out (s)	24.99	0.78	4.66
Turning (s)	17.19	1.5	4.81
Walking_in (s)	10.1	0.57	3.15
Turning_around (s)	11.43	1.5	3.73
Stand_to_Sit (s)	4	0.5	1.95
Sit To Stand			
Sit_to_Stand_Vert_Min_acc (m/s^2^)	4.53	0.26	1.83
Sit_to_Stand_Vert_Min_acc (m/s^2^)	−0.72	−4.12	−1.93
Sit_to_Stand_ML_Max_acc (m/s^2^)	2.26	0.1	0.86
Sit_to_Stand_ML_Min_acc (m/s^2^)	−0.34	−2.34	−0.94
Sit_to_Stand_AP_Max_acc (m/s^2^)	4.27	0.29	1.60
Sit_to_Stand_AP_Min_acc (m/s^2^)	−0.42	−2.69	−1.12
Sit_to_Stand_Extension_Peak (°)	60.2	4.9	33.36
Sit_to_Stand_Extension_Range (°)	39	0.1	14.21
Sit_to_Stand_Bending_Peak (°)	70	19.9	47.42
Sit_to_Stand_Bending_Range (°)	69.9	10.4	44.85
Turning			
First_Turn_Avg__Angular_Acc (m/s^2^)	88.4	11.4	43.76
First_turn_Peak_Angular_Acc (m/s^2^)	181.4	28.5	88.63
Turning Around			
Second_Turn_Avg_Angular_Acc (m/s^2^)	109.2	14.1	50.71
Second_turn_Peak_Angular_Acc (m/s^2^)	194.3	40.3	100.20
Stand To Sit			
Stand_to_Sit_Vert_Max_acc (m/s^2^)	9.84	0.39	4.88
Stand_to_Sit_Vert_Min_acc (m/s^2^)	−0.59	−4.84	−2.39
Stand_to_Sit_ML_Max_acc (m/s^2^)	3.69	0.67	1.78
Stand_to_Sit_ML_Min_acc (m/s^2^)	−0.53	−6.59	−1.87
Stand_to_Sit_AP_Max_acc (m/s^2^)	6.19	0.43	3.02
Stand_to_Sit_AP_Min_acc (m/s^2^)	0.11	−2.5	−0.98
Stand_to_Sit_Extension_Peak (°)	55.8	1	11.82
Stand_to_Sit_Extension_Range (°)	66.5	0.6	42.28
Stand_to_Sit_Bending_Peak (°)	75.5	19.5	53.27
Stand_to_Sit_Bending_Range (°)	62.6	0	24.94

**Table 2:** Average test performance results after 100 repetitions of hold-out cross-validation of different classifiers for sets of features extracted from the TUG test phases enumerated in Table 1.

Feature Set	Classifier	Accuracy	Sensitivity	Specificity
	RF	0.62	0.08	0.84
	SVM	0.65	0.25	0.80
6 Most Important Feature Set	KNN	0.69	0.04	0.95
	NB	0.65	0.25	0.80
	LR	0.66	0.04	0.90
	LD	0.67	0.04	0.92
	RF	0.60	0.17	0.77
	SVM	0.65	0.21	0.82
Phase Duration Features	KNN	0.65	0.46	0.72
	NB	0.66	0.21	0.84
	LR	0.66	0.13	0.87
	LD	0.66	0.13	0.87
	RF	0.68	0.17	0.89
	SVM	0.54	0.21	0.67
All Feature Set	KNN	0.58	0.33	0.67
	NB	0.59	0.25	0.72
	LR	0.48	0.21	0.59
	LD	0.57	0.36	0.62

**Table 3:** Average test performance results after 100 repetitions of hold-out cross-validation of different classifiers using the 20 most significant wavelet scattering features extracted from the acceleration magnitude signal recorded along the TUG test.

Classifier.	Accuracy	Sensitivity	Specificity	AUC
RF	0.81	0.10	0.99	0.75
SVM	0.69	0.29	0.79	0.64
KNN	0.77	0.10	0.94	0.61
NB	0.79	0.52	0.86	0.77
LR	0.71	0.19	0.84	0.61
LD	0.73	0.19	0.86	0.70

**Table 4:** Average test performance results after 100 repetitions of hold-out cross-validation for the 1D CNN architectures.

	1D CNN		
	RMSProp	SGDM	Adam
Accuracy	0.84	0.81	0.81
Sensitivity	0.33	0.33	0
Specificity	0.96	0.92	1
Precision	0.66	0.50	0
AUC	0.63	0.65	0.5

**Table 5:** Average test performance results after 100 repetitions of hold-out cross validation for the LSTM architectures.

	LSTM	LSTM	LSTM	BLSTM	BLSTM	BLSTM
	RMSProp	SGDM	Adam	RMSProp	SGDM	Adam
Accuracy	0.87	0.80	0.80	0.83	0.83	0.87
Sensitivity	0.33	0.16	0.16	0.33	0.50	0.33
Specificity	1	0.96	0.96	0.96	0.92	1
Precision	1	0.50	0.50	0.66	0.85	1
AUC	0.60	0.64	0.62	0.66	0.73	0.78

## Data Availability

Restrictions apply to the availability of these data. Data were obtained from Matia Fundazioa and are available from the authors with the permission of Matia Fundazioa.
